# Association between maternal blood lipids levels during pregnancy and risk of small-for-gestational-age infants

**DOI:** 10.1038/s41598-020-76845-1

**Published:** 2020-11-16

**Authors:** Qinqing Chen, Huiqi Chen, Fangfang Xi, Matthew Sagnelli, Baihui Zhao, Yuan Chen, Mengmeng Yang, Dong Xu, Ying Jiang, Guangdi Chen, Qiong Luo

**Affiliations:** 1 Department of Obstetrics, Women’s Hospital, Zhejiang University School of Medicine, No.1 Xueshi Road, Hangzhou, China; 2 Department of Public Health, Zhejiang University School of Medicine, 866 Yuhangtang Road, Hangzhou, China; 3 Department of Reproductive Endocrinology of Women’s Hospital, Zhejiang University School of Medicine, Hangzhou, China; 4 University of Connecticut School of Medicine, Farmington, CT USA

**Keywords:** Lipids, Risk factors

## Abstract

Dyslipidemia in pregnancy are associated with risk of adverse outcomes. As an adverse pregnancy outcome, small-for-gestational-age has been extensively studied in Western countries. However, similar studies have rarely been conducted in Asian countries. Data were derived from 5695 pairs of non-diabetic mothers and neonates between 1 Jan 2014 and 31 Dec 2014. 5.6% neonates in our study were SGA. Serum samples were collected during second and third trimesters for evaluation on fasting lipids levels. The present study intended to explore the associations between maternal lipid profile and small-for-gestational-age neonates. Odds ratios (ORs) and 95% confidence intervals (CIs) were calculated and adjusted via logistic regression analysis. After adjustments for confounders, third-trimester total cholesterol levels were associated with a decreased risk for small-for-gestational-age (aOR = 0.622, 95% CI 0.458–0.848, P = 0.002), and third-trimester high-density lipoprotein cholesterol and low-density lipoprotein cholesterol levels were associated with an increased risk for small-for-gestational-age (aOR = 1.955, 95% CI 1.465–2.578, P < 0.001; aOR = 1.403, 95% CI 1.014–1.944, P = 0.041).In the highest gestational weight gain strata, especially the third-trimester, the effect of high-density lipoprotein cholesterol levels on the risk for small-for-gestational-age is larger. High high-density lipoprotein cholesterol level during third trimester could be considered as indicators of a high-risk of small-for-gestational-age, regardless of gestational weight gain.

## Introduction

Small-for-gestational-age (SGA) is defined as birth weight below the 10th percentile of gestational age-specific standards according to an appropriate population^1–3^. SGA is considered one of the major complications of pregnancy, and is due to complex etiologies which can include maternal, fetal, placental, or external factors. The morbidity of SGA is about 6.39% in China. The mortality risk of perinatal infants with SGA is 6–9 times higher than infants with normal birth weight, and infants with SGA are at a rose risk for neonatal complications such as metabolic disorders, hypothermia, respiratory distress syndrome, necrotizing enterocolitis and retinopathy. SGA infants are at an increased risk for mortality secondary to metabolic diseases in adulthood including type 2 diabetes mellitus, obesity, hypertension, hyperlipidemia and insulin resistance^4,5^. Many cases of SGA show no abnormal findings during pregnancy. Ultrasound measurement is the main method to estimate fetal weight, but it often fails to predict SGA infants before they are born. The accuracy varies with gestational age, size of the baby, maternal condition and sonographer. Effective treatments for SGA are limited and it is difficult to reverse once SGA occurs. This highlights the need for emphasis on the prediction and prevention of SGA. However, current prediction methods have certain limitations, especially in low-risk pregnancies with no complications.

Well-established risk factors for SGA include primiparity, low pre-pregnancy body mass index (BMI), short stature, smoking and preeclampsia (PE)^6^. Recently, dyslipidemia in pregnancy has also been found to be associated with SGA and other adverse outcomes including gestational diabetes mellitus (GDM), PE, preterm birth. Many animal studies have demonstrated that maternal dyslipidemia affects the long-term health of the offsprings^7,8^ . A study also confirmed that the changes caused by maternal dyslipidemia in the perinatal period cannot be adjusted after birth^9^.

In a normal pregnancy,lipid parameters including total cholesterol (TC), triglycerides (TG), low-density lipoprotein-cholesterol (LDL-C), high-density lipoprotein-cholesterol (HDL-C) and phospholipid gradually increase starting in the 12th week of gestation and continue to do so through the second and third trimesters^10–13^. This increase in lipids sustains the demand of physiological changes and fetal growth. The accumulation of maternal fat depots and hyperlipidemia are the two principal changes in lipid metabolism during pregnancy^7^. Studies have shown that maternal dyslipidemia can predict the occurrence of pregnancy complications and adverse perinatal outcomes. When combined with maternal risk factors and other blood biochemical indexes, it has higher predictive value. Herrera et al. reported that maternal impaired TG and non-esterified fatty acid metabolism were correlated with excessive fetal growth^7^. The Amsterdam Born Children and their Development cohort study discovered that maternal TG concentrations in early pregnancy were linearly related with the prevalence of pregnancy-induced hypertension, PE, induced preterm birth and large for gestational age (LGA)^14^. Studies conducted in women diagnosed with GDM also showed that maternal TG and non-esterified fatty acid concentrations in late pregnancy are positively correlated with newborns’ birth weight, body mass index (BMI) and fat mass^15–17^.

Controversies still exist on whether the correlation between maternal dyslipidemia and neonatal birth weight appears only in GDM/ diabetic pregnancies or also in non-diabetic ones^15–19^. Therefore, we excluded pregnancies with GDM, diabetes, PE and other relevant complications in our study to attempt to provide some potential evidence for explaining the existing controversies. Similar studies have rarely been conducted before. Our study was aimed at deeply investigating the relationship between maternal dyslipidemia and SGA, with a focus on SGA pregnancies without other complications.

## Materials and methods

### Study population

Between 1 Jan 2014 and 31 Dec 2014, pregnant women who maintained regular prenatal healthcare and were planning on giving birth at Women’s Hospital, Zhejiang University School of Medicine were invited to participate in the study. Before enrollment, approval of the study was obtained from the Clinical Research Ethics Committee of Women’s hospital Zhejiang University School of medicine (the reference number: 20170160) and written informed consent was signed by every participant. We confirmed that all research was performed in accordance with Declaration of Helsinki. We established the study cohort based on inclusion and exclusion criteria. Inclusion criteria of pregnant women were: (1) maternal age at delivery between 19–44 years old; (2) had integrated medical records; and (3) singleton pregnancy. Exclusion criteria of pregnant women were: (1) had malignant tumor, diabetes mellitus, chromosomal abnormalities, inherited metabolic diseases before pregnancy; (2) experienced serious infection during early pregnancy; (3) used tobacco, consumed alcohol or drugs that affect blood lipid metabolism during pregnancy; and (4) pregnancy complications such as GDM, pregnancy-induced hypertension, preeclampsia and intrahepatic cholestasis of pregnancy (ICP).

All the women included were requested to complete a general medical record about sociodemographic characteristics, including maternal age, education background, gravidity, parity, height, pre-pregnancy weight, and other important information. Gestational age was calculated based on the last menstrual period and was confirmed by an ultrasonographic examination performed before 20 weeks of gestation. Fasting blood glucose (FBG) and lipid concentrations were assessed upon entry into the study, and pregnancy complications were documented from medical records during gestation. Information on maternal weight before delivery, delivery mode, gestational age, newborn sex, birth weight, Apgar scores and perinatal outcomes were recorded by midwives or obstetricians upon delivery and retrieved from medical records after delivery. Inclusion criteria for newborns were singleton and 5-min-postpartum Apgar scores ≥ 7. Exclusion criteria for newborns were preterm births (before 37 completed weeks) or expired delivery (more than 42 completed weeks), chromosomal abnormalities, inherited metabolic diseases and congenital abnormalities. In total, 5695 pairs of mothers and neonates were included in our study.

### Biochemical analyses

Venous blood samples for lipid assessment were taken after overnight fasting from all the participants at the second (24–26 gestational weeks) and third (30–32 gestational weeks) trimester of pregnancy. Every sample was assayed for TC, TG, HDL-C and LDL-C concentrations. TC and TG were assayed with the cholesterol oxidase-phenol aminophenazone method, and glycerol-3-phosphatase oxidase-phenol aminophenazone method, respectively. HDL-C and LDL-C were measured by homogeneous enzymatic colorimetric assays. All the lipid measurements were performed on an automatic biochemical analyser (Abbott Architect C16000, Abbott Laboratories, USA) respectively with TC, TG, HDL-C and LDL-C detection kits (Abbott Diagnostic Kit, Abbott Laboratories, USA).

### Definitions

BMI was calculated by dividing weight in kilograms by the square of height in meters. Maternal pre-pregnancy BMI was calculated from pre-pregnancy height and weight, and categorized into underweight (< 18.5 kg/m^2^), normal weight (18.5–24.9 kg/m^2^), overweight (25.0–29.9 kg/m^2^), and obese (≥ 30.0 kg/m^2^) groups on the basis of the World Health Organization BMI classification^20^. Gestational weight gain (GWG) was calculated as pre-pregnancy weight subtracted from the measured weight recorded at the last prenatal visit before delivery. According to the new recommendations from American Institute of Medicine, GWG was stratified into appropriate, inadequate and excessive groups^21^. Based on different pre-pregnancy BMIs, appropriate GWG was defined as 12.5–18.0 kg in underweight women, 11.5–16.0 kg in normal weight women, 7.0–11.5 kg in overweight women and 5.0–9.0 kg in obese women. Falling below the thresholds was defined as inadequate GWG, while exceeding the thresholds was defined as excessive GWG.

The World Health Organization (WHO) defined anemia during pregnancy as a haemoglobin concentration below 110 g/L at any time point in the pregnancy^22^. Newborns were classified into appropriate for gestational age (AGA), SGA and LGA on the basis of Neonatal Birth Weight for Gestational Age and Percentile in 23 Cities of China^23^. Infants with birth weight above 90th percentile for gestational age were classified as LGA, and small-for-gestational age (SGA) was defined as the lowest 10th percentile, those having weight between 10 and 90th percentile were appropriate for gestational age (AGA).

### Statistical analysis

Normally and non-normally distributed continuous variables were respectively presented as mean ± standard deviation (SD) and median (interquartile range, IQR), categorical variables were presented as N (%) in our study. Characteristics between SGA group and AGA group were compared using one-way ANOVA (for continuous variables) or χ^2^ test (for categorical variables). Serum TC, TG, LDL-C, HDL-C concentrations at the second and third trimesters between SGA group and AGA group were compared using Mann–Whitney test. Maternal lipid (TC, TG, LDL-C, HDL-C) increases from the second to third trimester were compared using Wilcoxon matched-pairs signed-ranks test. Logistic regression analysis was applied to explore the associations between maternal dyslipidemia and SGA. In the multivariable adjusted model, maternal age, marriage status, race/ethnicity, gravidity, parity, gestational age at birth, infant gender, height, pre-pregnancy weight, gestational weight gain, anemia, and FBG were regarded as confounding variables. All the analyses were performed with R version 3.4.3 for Windows (The R Project; https://www.r-project.org). P values < 0.05 were defined as statistically significant.

## Results

### Characteristics of the study subject

**Figure 1:**
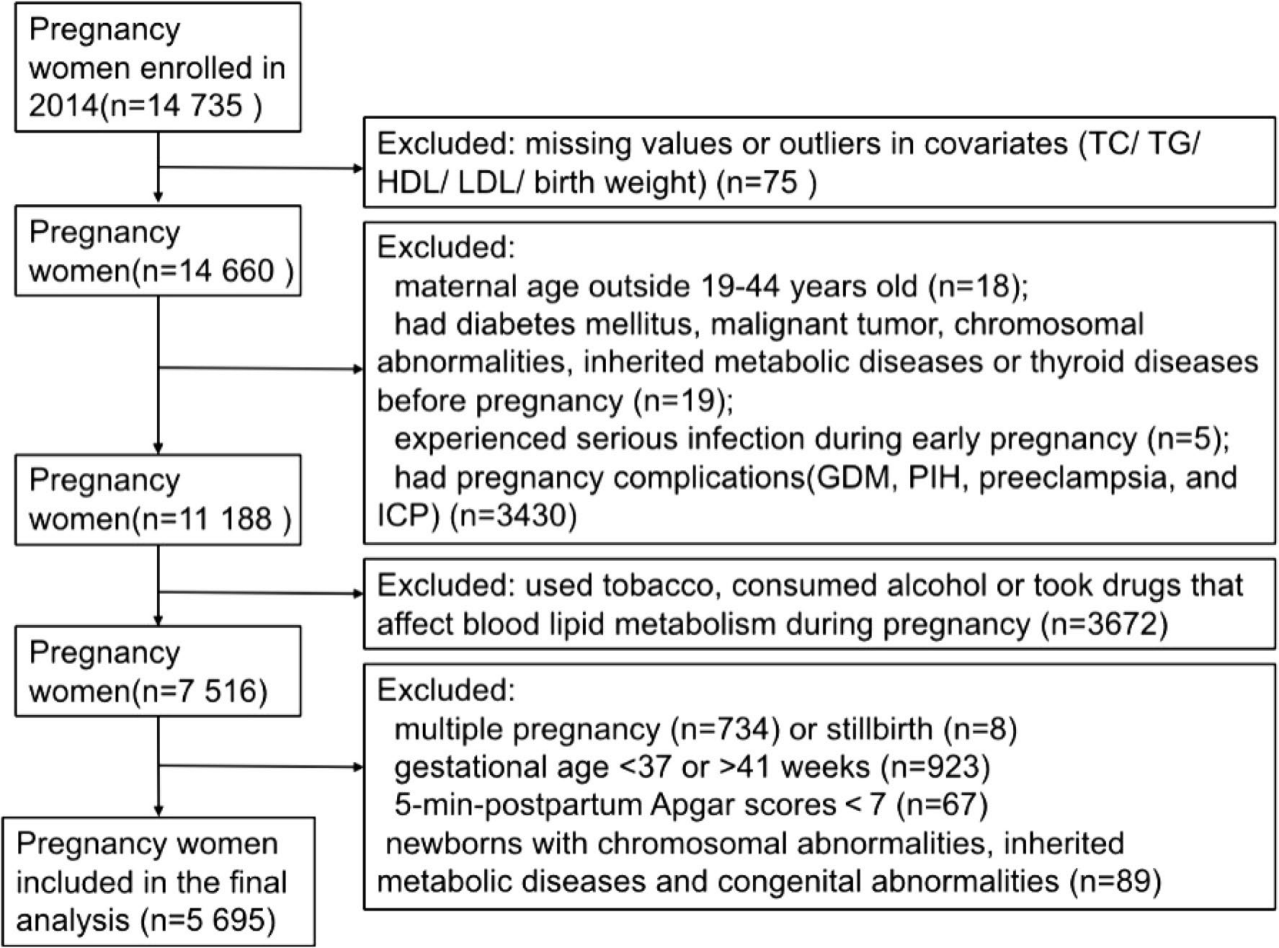
Flow diagram of study inclusion and exclusion process. In total, 14,735 patients were recruited in Women’s Hospital, Zhejiang University School of Medicine in 2014. Among them, 14,660 participants had complete data on lipid measurements and other covariates. We excluded 18 women who are young (< 19 years) or in elder age (> 44 years), 24 women who had diabetes mellitus, malignant tumor, inherited metabolic diseases before pregnancy, or experienced serious infection during early pregnancy, and 3430 women pregnancy complications such as gestational diabetes mellitus (GDM), pregnancy-induced hypertension (PIH), preeclampsia (PE) and intrahepatic cholestasis of pregnancy (ICP). We additionally excluded 3672 women who used tobacco, consumed alcohol or took anti-hyperlipidemic medication during pregnancy. After excluding newborn characteristics (not singleton, stillbirth, 5-min-postpartum Apgar scores < 7, premature or post-term delivery, or newborns with chromosomal abnormalities, inherited metabolic diseases and congenital abnormalities), the final analytic sample of 5695 participants was obtained.

The process of inclusion and exclusion was shown in Fig. 1, and the maternal and neonatal characteristics of our study population were shown in Table 1. Among the 5695 eligible mothers in the present study, the mean (SD) age at delivery was 28.78 (3.22) years old. Most of the women were nulliparous (n = 5094 [89.5%]), married (n = 5650 [99.2%]), Hans ethnicity (n = 5658 [99.4%]), had medical insurance (n = 5482 [96.3%]), and had a college-level education or higher (n = 5447 [95.7%]). About 4.5% of them were stratified as overweight or obese with pre-pregnancy BMI ≥ 25.0 kg/m^2^. Their mean (SD) pre-pregnancy BMI was 20.39 (2.52) kg/m^2^. The mean (SD) gestational weight gain was 14.81 (3.93) kg. According to the Institute of Medicine (IOM) recommendations for gestational weight gain, 53.9% met, 18.7% fell below and 27.4% exceeded the criteria. The newborns in our study had a mean (SD) birth weight of 3361.00 (385.94) g. 87.1% of them were AGA, 5.6% were SGA and 7.3% were LGA. The mean (SD) gestational age at birth was 39.69 (1.05) weeks. In addition, 2925 (51.4%) infants were boys.

**Table 1:** Characteristics of the study population.

Characteristics	Mean ± SD or n (%)
**Maternal characteristics**	n = 5695
Maternal age at delivery (years)	28.78 ± 3.22
Maternal height (m)	1.61 ± 0.05
Pre-pregnancy weight (kg)	52.94 ± 7.12
Weight before delivery (kg)	67.75 ± 7.82
**Pre-pregnancy BMI (kg/m**^**2**^**)**	20.39 ± 2.52
Underweight (< 18.5)	1294 (22.72)
Normal weight (18.5 to < 25.0)	4146 (72.80)
Overweight or obesity (≥ 25.0)	255 (4.48)
**Gestational weight gain (kg)**	14.81 ± 3.93
Inadequate	1062 (18.65)
Appropriate	3071 (53.92)
Excessive	1562 (27.43)
**Maternal education**	
Under college	248 (4.35)
College or equivalent	4724 (82.95)
Above college	723 (12.70)
**Medical insurance**	
Yes	5482 (96.26)
No	213 (3.74)
**Marital status**	
Married	5650 (99.21)
Others (Unmarried, divorced or widowed)	45 (0.79)
**Race**	
Hans	5658 (99.35)
Others	37 (0.65)
**Gravidity**	
1	3575 (62.77)
2	1452 (25.50)
≥ 3	668 (11.73)
**Parity**	
Nulliparous	5094 (89.45)
Multiparous	601 (10.55)
Anemia	1945 (34.15)
ALB (g/L)	38.40 ± 2.21
FBG (mmol/L)	4.39 ± 0.30
**Delivery mode**	
Vaginal delivery	1835 (32.22)
Cesarean section	3860 (67.78)
**Infant characteristics**	n = 5695
**Gender**	
Male	2925 (51.36)
Female	2770 (48.64)
Gestational age at birth (days)	277.83 ± 7.34
**Birth weight (g)**	3361.00 ± 385.94
< 2500	34 (0.60)
2500–4000	5331 (93.60)
≥ 4000	330 (5.80)
**Weight for gestational age**	
SGA	321 (5.64)
AGA	4961 (87.11)
LGA	413 (7.25)

*SD* standard deviance, *n* frequency, *%* proportion, *BMI* body mass index, *ALB* albumin, *FBG* Fasting blood glucose, *SGA/AGA/LGA* small/appropriate/large for gestational age.

### Comparison of characteristics and maternal lipid profile across SGA and AGA group

Table 2 shows maternal and neonatal characteristics of our study population across SGA and AGA group. Compared to AGA group, maternal height, pre-pregnancy BMI, gestational weight gain, marital status, parity and infant sex is significantly different in SGA group.

**Table 2:** Comparison of the maternal and neonatal characteristics across SGA and AGA group.

Variable, mean ± SD or n (%)	SGA		AGA	P value^†^
Total n	321		4961	
**Maternal characteristics**				
**Maternal age at delivery (years)**	28.60 ± 3.20		28.74 ± 3.18	0.471
< 25	21 (6.5)		318 (6.4)	0.360
25–29	202 (62.9)		2888 (58.2)	
30–34	83 (25.9)		1500 (30.2)	
≥ 35	15 (4.7)		255 (5.1)	
Maternal height (m)	1.60 ± 0.04		1.61 ± 0.05	< 0.001
Pre-pregnancy weight (kg)	49.68 ± 6.07		52.86 ± 7.03	< 0.001
Weight before delivery (kg)	63.26 ± 7.18		67.60 ± 7.64	< 0.001
**Pre-pregnancy BMI (kg/m**^**2**^**)**	19.47 ± 2.22		20.37 ± 2.50	< 0.001
Underweight (< 18.5)	109 (34.0)		1141 (23.0)	< 0.001
Normal weight (18.5 -24.9)	207 (64.5)		3611 (72.8)	
Overweight or obesity (≥ 25.0)	5 (1.6)		209 (4.2)	
**Gestational weight gain (kg)**	13.58 ± 3.31		14.75 ± 3.90	< 0.001
Inadequate	96 (29.9)		929 (18.7)	< 0.001
Appropriate	179 (55.8)		2719 (54.8)	
Excessive	46 (14.3)		1313 (26.5)	
**Education**				0.867
Under college	15 (4.7)		220 (4.4)	
College or equivalent	268 (83.5)		4456 (82.7)	
Above college	38 (11.8)		636 (12.8)	
**Medical insurance**				0.391
Yes	306 (95.3)		4776 (96.3)	
No	15 (4.7)		185 (3.7)	
**Marital status**				0.002
Married	313 (97.5)		4925 (99.3)	
Others (Unmarried, divorced or widowed)	8 (2.5)		36 (0.7)	
**Race**				0.120
Hans	316 (98.4)		4929 (99.4)	
Others	5 (1.6)		32 (0.6)	
**Gravidity**				0.042
1	228 (71.0)		3148 (63.5)	
2	74 (23.1)		1240 (25.0)	
≥ 3	19 (0.3)		970 (19.5)	
**Parity**				< 0.001
Nulliparous	307 (95.6)		4446 (89.6)	
Multiparous	14 (4.4)		515 (10.4)	
Anemia	87 (27.1)		1708 (34.4)	0.007
ALB (g/L)	38.48 ± 2.31		38.43 ± 2.19	0.694
FBG (mmol/L)	4.31 ± 0.31		4.39 ± 0.30	< 0.001
**Infant characteristics**				
**Gender**				0.001
Male	116 (36.1)		2537 (51.1)	
Female	205 (63.9)		2424 (48.9)	
Gestational age at birth (days)	277.71 ± 6.95		277.70 ± 7.34	0.981
Birth weight (g)	2681.18 ± 160.22		3342.53 ± 295.67	< 0.001

*BMI* body mass index, *ALB* albumin, *FBG* Fasting blood glucose. *SGA/AGA* small/appropriate for gestational age.

^†^P values were calculated using one-way ANOVA (for continuous variables) or χ^2^ test (for categorical variables), and P < 0.05 indicates that the mean values (for continuous variables) or proportions (for categorical variables) of a variable were significantly different between SGA group and AGA group.

Table 3 shows maternal lipid profile by trimester. 4 lipid parameters (TC, TG, LDL-C and HDL-C) in the second trimester and TG in the third trimester were lower in SGA group while HDL-C in the third trimester was higher in SGA group. In addition, in the SGA and AGA groups, serum TC, TG, LDL-C levels were increased in the third trimester compared to the second trimester, while HDL-C decreased as pregnancy advanced (p < 0.001) (Supplementary Table S2). Supplementary Table S1 shows maternal lipid profile in percentiles by trimester.

**Table 3:** Comparison of maternal lipid profile by trimester between SGA and AGA group.

Trimester	SGA	AGA	P value#
**Second**			
TC	5.94 (5.36–6.58)	6.16 (5.52–6.85)	< 0.001
TG	1.87 (1.51–2.43)	2.05 (1.62–2.58)	< 0.001
HDL-C	2.29 (2.02–2.57)	2.32 (2.05–2.78)	0.048
LDL-C	3.20 (2.72–3.76)	3.36 (2.85–3.91)	0.003
**Third**			
TC	6.59 (5.77–7.38)	6.64 (5.85–7.46)	0.546
TG	2.72 (2.20–3.59)	3.05 (2.40–4.00)	< 0.001
HDL-C	2.17 (1.88–2.59)	2.07 (1.78–2.38)	< 0.001
LDL-C	3.68 (2.98–4.33)	3.63 (3.02–4.30)	0.943

*SGA/AGA* small/appropriate for gestational age, *TC* total cholesterol, *TG* triglycerides, *LDL-C/HDL-C* low-density/high density lipoprotein cholesterol-cholesterol.

Maternal lipid levels and increases were presented as median (IQR) mmol/L.

^#^P values were calculated using Mann–Whitney test, and P < 0.05 indicates that the median values of lipid variables were significantly different between SGA group and AGA group.

### Associations between maternal lipid profile and SGA

Tables 4 and 5 display the associations between maternal second- and third-trimester lipid profile and SGA. In our study, the incidence of SGA and LGA was 5.6% and 7.3%, respectively. Table 4 showed a positive association between third-trimester HDL-C and SGA, a negative association between second-trimester lipids (TC, TG, LDL-C and HDL-C), third-trimester TG and SGA, and no significant association between third-trimester TC or LDL-C with SGA. However, Table 5 demonstrates different associations between maternal lipid levels and SGA. Multivariate analysis revealed that when adjusted for maternal age, race, marital status, gravidity, parity, height, weight before pregnancy, gestational weight gain, infant gender, anemia, and FBG, third-trimester TC level was associated with a decreased risk for SGA (aOR = 0.622, 95% CI 0.458–0.848, p = 0.002), and third-trimester HDL-C and LDL-C levels were associated with an increased risk for SGA (aOR = 1.955, 95% CI 1.465–2.578, p < 0.001; aOR = 1.403, 95% CI 1.014–1.944, p = 0.041). In contrast, there were no significant associations between second-trimester TC, TG, HDL-C and LDL-C levels and SGA in multivariate analysis. In addition, associations of blood lipids with SGA were kept robust when participants with LGA were included (Table 6).

**Table 4:** Association between maternal and neonatal characteristics and the risk of SGA in univariate logistic regression analysis.

Variates	Unadjusted OR	95%CI of OR		P value
		LL	UL	
Anemia	0.708	0.547	0.908	0.007
Above college or equivalence (v. below college)	0.928	0.700	1.225	0.602
Medical insurance	0.790	0.477	1.412	0.392
Married (v. unmarried, divorced or widowed)	0.286	0.139	0.667	0.002
Hans ethnicity (v. other ethnicity)	0.410	0.173	1.207	0.066
Maternal age at birth	0.410	0.173	1.207	0.066
Gravidity	0.742	0.623	0.871	< 0.001
Multiparous (v. nulliparous)	0.394	0.218	0.652	0.001
Height (cm)	0.937	0.913	0.961	< 0.001
Pre-pregnancy weight	0.927	0.910	0.945	< 0.001
Gestational weight gain	0.920	0.891	0.949	< 0.001
Weight before delivery	0.919	0.904	0.935	< 0.001
ALB	1.010	0.960	1.064	0.694
FBG	0.384	0.264	0.560	< 0.001
Gestational age at delivery	1.000	0.985	1.016	0.982
Female infant	1.850	1.466	2.344	< 0.001
**Second**				
TC	0.823	0.734	0.923	0.001
TG	0.803	0.684	0.935	0.006
HDL-C	0.771	0.611	0.967	0.026
LDL-C	0.843	0.732	0.969	0.017
**Third**				
TC	0.976	0.894	1.038	0.557
TG	0.845	0.767	0.926	< 0.001
HDL-C	1.534	1.268	1.846	< 0.001
LDL-C	1.012	0.904	1.132	0.828

*OR* odds ratio, *CI* confidence interval, *ALB* albumin, *FBG* fasting blood glucose, *TC* total cholesterol, *TG* triglycerides, *LDL-C/HDL-C* low-density/high-density lipoprotein cholesterol-cholesterol, *SGA* small for gestational age.

**Table 5:** Association between blood lipids and the risk of SGA in multivariate logistic models.

Lipids	Unadjusted			Model 1^a^		Model 2^b^		Model 3^c^	
	OR (95%CI)	P value		aOR (95%CI)	P value	aOR (95%CI)	P value	aOR (95%CI)	P value
**Second**									
TC	0.823 (0.734–0.923)		0.001	0.878 (0.663–1.278)	0.440	0.819 (0.621–1.200)	0.235	0.796 (0.610–1.153)	0.156
TG	0.803 (0.684–0.935)		0.006	0.878 (0.728–1.045)	0.159	0.922 (0.762–1.099)	0.386	0.933 (0.772–1.111)	0.454
HDL-C	0.771 (0.611–0.967)		0.026	0.760 (0.518–1.065)	0.136	0.825 (0.559–1.153)	0.295	0.838 (0.572–1.167)	0.330
LDL-C	0.843 (0.732–0.969)		0.017	1.005 (0.683–1.327)	0.979	1.029 (0.697–1.350)	0.867	1.078 (0.739–1.398)	0.644
**Third**									
TC	0.976 (0.894–1.038)		0.557	0.671 (0.492–0.911)	0.011	0.641 (0.469–0.875)	0.005	0.622 (0.458–0.848)	0.002
TG	0.845 (0.767–0.926)		< 0.001	0.995 (0.879–1.099)	0.939	1.023 (0.902–1.122)	0.708	1.043 (0.925–1.134)	0.423
HDL-C	1.534 (1.268–1.846)		< 0.001	1.841 (1.380–2.439)	< 0.001	1.934 (1.446–2.566)	< 0.001	1.955 (1.465–2.578)	< 0.001
LDL-C	1.012 (0.904–1.132)		0.828	1.397 (1.013–1.938)	0.044	1.345 (0.971–1.869)	0.077	1.403 (1.014–1.944)	0.041

*aOR* adjusted odds ratio, *CI* confidence interval, *TC* total cholesterol, *TG* triglycerides, *LDL-C/HDL-C* low-density/high density lipoprotein cholesterol-cholesterol, *SGA* small for gestational age.

^a^Adjusted for maternal age, marriage status, race, gravidity, parity, gestational age at birth, and infant gender.

^b^Model 2 is model 1 plus adjustment for maternal height, weight before pregnancy, and gestational weight gain.

^c^Model 3 is model 2 plus adjustment for anemia and FBG.

**Table 6:** Association between blood lipids and the risk of SGA in multivariate logistic models among pregnant women.

Lipids	Unadjusted			Model 1^a^		Model 2^b^		Model 3^c^	
	OR (95%CI)	P value		aOR (95%CI)	P value	aOR (95%CI)	P value	aOR (95%CI)	P value
**Second**									
TC	0.824 (0.735 -0.923 )		0.001	0.885 (0.666–1.297)	0.477	0.819 (0.618–1.209)	0.246	0.794 (0.607–1.156)	0.157
TG	0.776 (0.661 -0.905 )		0.002	0.857 (0.710–1.021)	0.096	0.909 (0.750–1.085)	0.309	0.919 (0.759–1.095)	0.364
HDL-C	0.802 (0.637 -1.002 )		0.056	0.772 (0.524–1.085)	0.164	0.836 (0.564–1.172)	0.336	0.851 (0.579–1.186)	0.376
LDL-C	0.838 (0.728 -0.962 )		0.013	0.990 (0.669–1.309)	0.956	1.021 (0.687–1.341)	0.906	1.072 (0.732–1.390)	0.669
**Third**									
TC	0.979 (0.897 -1.040 )		0.592	0.668 (0.491–0.904)	0.010	0.637 (0.466–0.869)	0.005	0.615 (0.453–0.838)	0.002
TG	0.825 (0.748 -0.904 )		< 0.001	0.983 (0.867–1.092)	0.781	1.013 (0.892–1.118)	0.831	1.036 (0.916–1.130)	0.529
HDL-C	1.604 (1.329 -1.928 )		< 0.001	1.903 (1.427–2.524)	< 0.001	1.982 (1.481–2.636)	< 0.001	2.005 (1.500–2.651)	< 0.001
LDL-C	1.021 (0.912 -1.140 )		0.719	1.400 (1.018–1.938)	0.041	1.348 (0.974–1.871)	0.074	1.412 (1.020–1.954)	0.038

Pregnant women who conceived LGA infant (n = 413) were included in the data analyses.

*aOR* adjusted odds ratio, *CI* confidence interval, *TC* total cholesterol, *TG* triglycerides, *LDL-C/HDL-C* low-density/high-density lipoprotein cholesterol-cholesterol.

^a^Adjusted for maternal age, marriage status, race, gravidity, parity, gestational age at birth, and infant gender.

^b^Model 2 is model 1 plus adjustment for maternal height, weight before pregnancy, and gestational weight gain.

^c^Model 3 is model 2 plus adjustment for anemia and FBG.

We further explored the relationship between HDL-C and SGA. Table 7 shows that the highest quintile of third-trimester HDL-C were at an increased risk of SGA, which demonstrates that dyslipidemia (high HDL-C) is a risk factor of SGA. Table 8 shows that the association of third-trimester HDL-C levels with increased risk for SGA rises across different gestational weight gain strata (Inadequate GWG: aOR = 1.567, 95% CI 1.022–2.607; Appropriate GW: aOR = 1.900, 95% CI 1.301–2.738; Excessive GWG: aOR = 2.525, 95% CI 1.217–5.235).

**Table 7:** Association between third-trimester HDL-C and the risk of SGA in multivariate logistic models.

Lipid	All	SGA, n (%)	Unadjusted OR (95% CI)	P value	aOR (95% CI)^a^	P value
**HDL-C of 3rd trimester**						
Q1: < 1.71	1007	43 (4.3)	1		1	
Q2: 1.71–1.94	996	58 (18.1)	1.386 (0.925–2.077)	0.114	1.312 (0.868–1.983)	0.197
Q3: 1.95–2.17	1076	62 (19.3)	1.371 (0.920–2.043)	0.121	1.249 (0.830–1.878)	0.286
Q4: 2.18–2.49	1103	62 (19.3)	1.335 (0.896–1.989)	0.155	1.139 (0.757–1.712)	0.532
Q5: > = 2.50	1100	96 (29.9)	2.144 (1.480–3.104)	< 0.001	1.909 (1.303–2.796)	0.001

*aOR* adjusted odds ratio, *CI* confidence interval, *TC* total cholesterol, *TG* triglycerides, *LDL-C/HDL-C* low-density/high-density lipoprotein cholesterol-cholesterol, *SGA* small for gestational age.

^a^Adjusted for maternal age, marriage status, race, gravidity, parity, gestational age at birth, infant gender, maternal height, weight before pregnancy, gestational weight gain, anemia and FBG.

**Table 8:** Associations between maternal blood lipids and risk of SGA across different gestational weight gain strata.

Lipid	Unadjusted			Inadequate GWG		Appropriate GWG		Excessive GWG	
	OR (95%CI)	P value		aOR (95%CI) ^d^	P value	aOR (95%CI) ^d^	P value	aOR (95%CI)^a^	P value
**Second**									
TC	0.823 (0.734–0.923)		0.001	0.603 (0.225–1.672)	0.332	0.898 (0.629–1.582)	0.645	0.753 (0.427–1.713)	0.400
TG	0.803 (0.684–0.935)		0.006	1.014 (0.671–1.470)	0.944	0.900 (0.687–1.148)	0.421	0.920 (0.559–1.392)	0.719
HDL-C	0.771 (0.611–0.967)		0.026	1.147 (0.444–2.661)	0.770	0.642 (0.360–1.030)	0.094	1.205 (0.527–2.314)	0.619
LDL-C	0.843 (0.732–0.969)		0.017	1.316 (0.474–3.525)	0.596	1.033 (0.586–1.427)	0.889	1.108 (0.450–2.304)	0.799
**Third**									
TC	0.976 (0.894–1.038)		0.557	0.995 (0.757–1.088)	0.958	0.496 (0.333–0.741)	0.001	0.545 (0.236–1.078)	0.156
TG	0.845 (0.767–0.926)		< 0.001	1.022 (0.847–1.260)	0.816	1.060 (0.919–1.156)	0.283	0.891 (0.619–1.243)	0.518
HDL-C	1.534 (1.268–1.846)		< 0.001	1.567 (1.022–2.607)	0.042	1.900 (1.301–2.738)	0.001	2.525 (1.217–5.235)	0.013
LDL-C	1.012 (0.904–1.132)		0.828	0.824 (0.628–1.381)	0.215	1.904 (1.241–2.935)	0.003	1.567 (0.697–3.818)	0.319

*aOR* adjusted odds ratio, *CI* confidence interval, *TC* total cholesterol, *TG* triglycerides, *LDL-C/HDL-C* low-density/high-density lipoprotein cholesterol-cholesterol, *GWG* gestational weight gain, *SGA* small for gestational age.

^a^Adjusted for maternal age, marriage status, race, gravidity, parity, gestational age at birth, infant gender, maternal height, weight before pregnancy, anemia and FBG.

Tables S3 and S4 display the associations between maternal second- and third-trimester lipid levels and SGA, respectively. Specifically, multivariate analysis revealed that when adjusted for maternal age, race, marital status, gravidity, parity, and infant gender, second-trimester TG level was associated with a decreased risk for SGA (aOR = 0.766, 95% CI 0.638–0.909, p = 0.003) , while there were no significant associations between second-trimester TC, HDL-C and LDL-C levels and SGA. After adusted for the same confounders in multivariate analysis, third-trimester TC level was associated with a decreased risk for SGA (aOR = 0.658, 95% CI 0.493–0.873, p = 0.004), and third-trimester HDL-C and LDL-C levels were associated with an increased risk for SGA (aOR = 1.792, 95% CI 1.371–2.341, p < 0.001; aOR = 1.479, 95% CI 1.099–2.004, p = 0.011) (just similar with the result showed in Table 5).

We have summarized published studies evaluating the associations between maternal serum lipids and neonatal birth weight or the risk of SGA at different gestational periods (Table S5). Most evidence suggests that second-trimester TG and third-trimester HDL-C increase the risk of SGA infants, but TG in the third trimester is a protective factor.

## Discussion

In our population-based study, we comprehensively explored the relationship between maternal lipid concentrations and SGA in Chinese reproductive-age women without pregnancy complications. During pregnancy, the ability of intestinal absorption of fat increased and is primarily driven by hormonal changes. As the pregnancy progresses, serum levels of TG, TC, LDL-C rose to store additional fat required for maintaining pregnancy, fetal growth and lactation, which was seen in our study as demonstrated by the increase of serum TC, TG, LDL-C levels in the third trimester compared to the second trimester. Placental trophoblast and endothelial cells can effectively transfer maternal cholesterol to the fetus throughout pregnancy, thus affecting on fetal growth and infant birth weight. The present result confirmed that high concentrations of HDL-C and LDL-C in the third trimester were significantly associated with an increased risk of SGA, which was consistent with two previous studies^24,25^. Furthermore, we found that the effect of third-trimester HDL-C levels associated with increased risk for SGA was rising across different gestational weight gain strata. At the same time, low maternal TC concentrations in the third-trimester were positively associated with the risk of SGA, which may be connected with malnutrition. These were the main findings of our study, suggesting that maternal dyslipidemia is an important risk factor of SGA and has an important impact on the metabolic mechanisms in pregnancy.

Association between maternal lipids and birth weight has been examined in previous studies among different races and countries. Jin et al^25^ found that the high third-trimester TG concentration was associated with a decreased risk for SGA and an increased risk for LGA. Di Cianni et al^28^ and a recent Dutch study^14^ also found similar results in different gestational ages. In recent years, studies have found that elevated TG levels lead to vascular endothelial dysfunction and enhanced lipid peroxidation which resulted in damage to vascular endothelial cells. In a normal pregnancy, the level of antioxidant system activity increases at the same time to resist lipid peroxidation, and endothelium-dependent relaxation also enhances in order to protect the pregnant women’s cardiovascular system during pregnancy. Moreover, HDL-C is also protective of vascular endothelial cells by removing fat from tissues. As a result, abnormal lipid peroxidation will not appear in a normal pregnancy. SGA is often found to be related to reduced vascular endothelial growth factors and placental apoptosis. Those pathological changes in the ultrastructure of placental tissues in SGA patients are connected with their blood lipid peroxide (LPO) levels. This may explain how disorders of lipid metabolism lead to SGA. Relatively lower levels of HDL and higher levels of TG prompted the vascular pathologies and pathological changes in ultrastructure of placental tissues. However, no significant result was found in regards to TG levels in our study, and results for HDL-C levels were contrary with this theory. Our findings regarding HDL levels seem to be counterintuitive given that high maternal HDL-C levels should be associated with reduced risk of adverse outcomes.

Consistent with our results, Michael et al.^24^ reported that mothers of SGA cases had significantly higher concentrations of HDL. In Clausen’s^26^ study, elevated second trimester serum HDL cholesterol concentrations were significantly associated with reduced risk of delivering a macrosomic (> 4500 g) infant. Misra et al.^27^ also reported a negative association between HDL-C and birth weight at all time points starting at 10 weeks’ gestation in overweight or obese women. One recent study^28^ showed that maternal serum HDL-C concentrations were inversely associated with birthweight at 24th and 36th gestational weeks and the high concentrations of HDL-C at the 36th gestational week increased the risk of SGA. There was a tendency that pregnant women with higher HDL-C concentrations throughout pregnancy gave birth to infants with lower birth weight. Trophoblasts absorb glycerol and freefatty acids released by placental enzyme-catalyzed hydrolysis of HDL and LDL and re-esterify them to the fetal development. HDL-C plays an important role in the conversion. In non-pregnant populations, HDL-C were known to be protctive against cardiovascular disease. However, in diseases states, normal HDL converts to dysfunctional HDL which regulates vascular endothelial cell function differently^29^. Studies have reported that the concentrations of HDL-C were related to pregnancy outcomes and complications, like spontaneous preterm delivery^30^. Previous research^31^ has found reduced cord blood HDL cholesterol levels in SGA infants. The elevated HDL-C concentration were more likely a consequence of the placental dysfunction, which may affect the transportation of maternal HDL across the placenta to the fetus and led to SGA. Other mechanisms may be also involved in the proccess of SGA. No cord blood levels of these lipids were available in our study, thus preventing us from investigating whether the placental dysfunction is responsible for the elevated maternal HDL-C concentrations and is reflected by reduced concentrations in the fetus. Collecting cord blood for lipid testing will be part of the next step of our investigation into the relationship between dyslipidemia and birth weight. One interesting findings of our study was that the elevation in third-trimester HDL-C concentration was associated with increased risk for SGA in women with appropriate gestational weight gain. This association suggested that HDL-C concentrations combined with gestational weight gain may be an underlying predictor of SGA.

Our study raised another controversy that maternal high LDL-C levels in the third-trimester were associated with an increased risk of SGA, which was unlike the results of previous studies. In Pecks’s study^32,33^, pregnancies with intrauterine fetal growth retardation were associated with lower LDL-C concentrations. Serizawa et al.^34^ indicated that lower maternal LDL-C levels in the second trimester were associated with an increased risk of delivering an SGA infant at term. Morteza et al.^35^ also found the same relationship and highlighted that intrauterine fetal growth restricted pregnancies were related to insulin resistance. They concluded that the hormonal imbalance underlying insulin resistance complicates intrauterine growth restricted pregnancies by reducing the consumption of LDL-C and lowering the triglyceride levels. In our study, we excluded women with GDM and diabetes, and the results of oral glucose tolerance test were within normal range for the study participants. Thus, we believe that our results were more reliable since there were likely no hormonal imbalances underlying insulin resistance. However, these mechanisms need to be further explored.

Our study has provided attractive evidence regardings the association of maternal lipid levels and SGA. There still exist many controversies surrounding the understanding and impact of lipid metabolism in pregnancy. Few studies have investigated dyslipidemia in SGA, especially in Asian countries, and less attention has been paid to HDL-C and LDL-C levels. Our study had a large sample size and excluded women with pregnancy complications. Hence, we believe that our results have less confounding factors and are a good complement to existing researches.

However, the present study still has some limitations. There is outlier in birth weight and missing values in lipid measurements. However, because of our large sample, we think this would have minor influence on our results, although we cannot rule out some residual confounding. We were not able to adjust for physical activity during pregnancy or family history of gestational diabetes, two factors that could confound our results. Our study collected the serum in the second trimester (24th–26th gestational age) and third trimester (30th–32th gestational age), which was suggested to be better to collect the maternal lipids concentrations across the whole pregnancy and even before pregnancy. No placental pathology was available for further investigation into the mechanisms behind the associations discussed in this study.

In spite of the striking discoveries in our study, it is still unlikely to be clinically used to predict the birth of SGA infants. Further research should be conducted in multiple centers, tracking the whole pregnancy to further illustrate the association between maternal lipids and SGA infants and to uncover the point in gestation that these differences manifest. The results of our study would warrant a multi-center and multi-region prospective investigation in future to establish a definition for gestational maternal dyslipidemia and its association with pregnancy outcomes and long term metabolic syndrome risk. More investigation into the underlying physiology and molecular mechanisms of these relationships need to be conducted to lend more reliability to the proposed markers in this study.

## Conclusions

Elevated maternal HDL-C and LDL-C levels measured during third trimester are risk factor for SGA, and high TC level during third trimester is inversely associated with SGA. High HDL level during third trimester could be considered as indicators of a high-risk of SGA, regardless of gestational weight gain.

## Supplementary information

Supplementary Information

## Data Availability

The datasets generated and analysed during the current study are available from the corresponding author on reasonable request.
