# Supplementary material for: Association between maternal blood lipids levels during pregnancy and risk of small-for-gestational-age infants
Source: Sci Rep. 2020 Nov 16;10:19865. doi: 10.1038/s41598-020-76845-1 (PMC7669834; doi:10.1038/s41598-020-76845-1)
Supplement: Supplementary file 1 — Supplementary Information [file 41598_2020_76845_MOESM1_ESM.docx]

**Association between maternal blood lipids levels during pregnancy and risk of small-for-gestational-age infants**

Qinqing Chen^1^, Huiqi Chen^2^, Fangfang Xi , Matthew Sagnelli, Baihui Zhao, Yuan chen, Mengmeng Yang, Dong Xu, Ying Jiang, Guangdi Chen*, Qiong Luo*

^1,2^These two authors contribute equally to this manuscript.

**Corresponding authors**: Qiong Luo, Department of obstetrics, Women’s Hospital, Zhejiang University School of Medicine, No.1 Xueshi Road, Hangzhou, China. E-mail: [luoq@zju.edu.cn](mailto:luoq@zju.edu.cn) or Guangdi Chen, Department of Public Health, Zhejiang University School of Medicine, 866 Yuhangtang Road, Hangzhou, China. Email: chenguangdi@zju.edu.cn.

| **Table S1** Maternal lipid profile by trimester | | | | | | | | | |
| --- | --- | --- | --- | --- | --- | --- | --- | --- | --- |
| Lipid parameters | Percentiles | | | | | | | | |
|  | 2.5 | 5 | 10 | 25 | 50 | 75 | 90 | 95 | 97.5 |
| Second |  |  |  |  |  |  |  |  |  |
| TC | 4.38 | 4.67 | 4.98 | 5.51 | 6.15 | 6.83 | 7.53 | 7.97 | 8.34 |
| TG | 1.06 | 1.21 | 1.34 | 1.63 | 2.06 | 2.60 | 3.21 | 3.68 | 4.16 |
| HDL-C | 1.55 | 1.66 | 1.79 | 2.04 | 2.32 | 2.76 | 3.08 | 3.25 | 3.42 |
| LDL-C | 1.91 | 2.17 | 2.43 | 2.84 | 3.36 | 3.91 | 4.47 | 4.85 | 5.17 |
| Third |  |  |  |  |  |  |  |  |  |
| TC | 4.58 | 4.87 | 5.22 | 5.84 | 6.63 | 7.46 | 8.28 | 8.93 | 9.42 |
| TG | 1.55 | 1.71 | 1.94 | 2.40 | 3.06 | 4.02 | 5.13 | 5.97 | 7.14 |
| HDL-C | 1.28 | 1.40 | 1.52 | 1.77 | 2.06 | 2.37 | 2.91 | 3.15 | 3.35 |
| LDL-C | 1.98 | 2.19 | 2.51 | 3.01 | 3.62 | 4.30 | 5.02 | 5.49 | 5.92 |

Abbreviations: TC, total cholesterol; TG, triglycerides; LDL-C/HDL-C, low-density/high-density lipoprotein cholesterol-cholesterol.

| **Table S2** Maternal lipid profile by trimester across SGA, AGA and LGA group | | | | | | | | | | | | | |  |
| --- | --- | --- | --- | --- | --- | --- | --- | --- | --- | --- | --- | --- | --- | --- |
| Lipids(mmol/L) | TC | | | TG | | | HDL-C | | | LDL-C | | | | |
| Trimester | 2nd vs 3rd | | P | 2nd vs 3rd | | P | 2nd vs 3rd | | P | 2nd vs 3rd | | P |  |  |
| All participants | 6.15(5.51-6.83) | 6.63(5.84-7.46) | <0.001 | 2.06(1.63-2.60) | 3.06(2.40-4.02) | <0.001 | 2.32(2.04-2.76) | 2.06(1.77-2.37) | <0.001 | 3.36(2.84-3.91) | 3.62(3.01-4.30) | <0.001 |  |  |
| SGA | 5.94(5.36-6.58) | 6.59(5.77-7.38) | <0.001 | 1.87(1.51-2.43) | 2.72(2.20-3.59) | <0.001 | 2.29(2.02-2.57) | 2.17(1.88-2.59) | 0.001 | 3.20(2.72-3.76) | 3.68(2.98-4.33) | <0.001 |  |  |
| AGA | 6.16(5.52-6.85) | 6.64(5.85-7.46) | <0.001 | 2.05(1.62-2.58) | 3.05(2.40-4.00) | <0.001 | 2.32(2.05-2.78) | 2.07(1.78-2.38) | <0.001 | 3.36(2.85-3.91) | 3.63(3.02-4.30) | <0.001 |  |  |
| LGA | 6.17(5.52-6.87) | 6.52(5.70-7.48) | <0.001 | 2.30(1.81-2.90) | 3.60(2.85-4.60) | <0.001 | 2.24(1.94-2.56) | 1.90(1.65-2.19) | <0.001 | 3.41(2.90-4.01) | 3.55(2.82-4.28) | <0.001 |  |  |

Abbreviations: TC, total cholesterol; TG, triglycerides; LDL-C/HDL-C, low-density/high-density lipoprotein cholesterol-cholesterol.

Maternal lipid levels were presented as median (IQR) mmol/L.

# P values were derived from the comparisons among the median values of lipid variables of the second and third trimesters using Wilcoxon matched-pairs signed-ranks test.

**Table S3** Associations between maternal second-trimester lipid levels and SGA

|  | Estimate | Std. error | aOR（95%CI）^a^ | P value |
| --- | --- | --- | --- | --- |
| Marriage | -1.088263 | 0.428352 | 0.337(0.154-0.847) | 0.011 |
| Race | -0.72764 | 0.490765 | 0.483 (0.201-1.434) | 0.138 |
| Age | 0.032241 | 0.017821 | 1.033 (0.997-1.069) | 0.070 |
| Gravidity | -0.204354 | 0.085117 | 0.815 (0.686-0.958) | 0.016 |
| Parity | -0.724133 | 0.302352 | 0.485 (0.259-0.853) | 0.017 |
| Gestational age at delivery | -0.001447 | 0.007421 | 0.999 (0.984-1.013) | 0.845 |
| Infant Gender | 0.590182 | 0.108658 | 1.804 (1.460-2.236) | <0.001 |
| TC | -0.032897 | 0.172234 | 0.968 (0.725-1.400) | 0.849 |
| TG | -0.266696 | 0.090277 | 0.766 (0.638-0.909) | 0.003 |
| HDL-C | -0.219056 | 0.176132 | 0.803 (0.558-1.108) | 0.214 |
| LDL-C | -0.053708 | 0.175852 | 0.948 (0.650-1.263) | 0.760 |

Abbreviations: SGA, small for gestational age; TC, total cholesterol; TG, triglycerides; LDL-C/HDL-C, low-density/high density lipoprotein cholesterol-cholesterol.

a: Adjusted for maternal age, race, marital status, gravidity, parity, and infant gender.

**Table S4** Associations between maternal third-trimester lipid levels and SGA

|  | Estimate | Std. error | aOR（95%CI）^a^ | P value |
| --- | --- | --- | --- | --- |
| Marriage | -1.110346 | 0.430382 | 0.329 (0.150-0.831) | 0.010 |
| Race | -0.837941 | 0.493748 | 0.433 (0.179-1.290) | 0.090 |
| Age | 0.035201 | 0.017939 | 1.036 (1.000-1.073) | 0.050 |
| Gravidity | -0.192865 | 0.084791 | 0.825 (0.6884-0.968) | 0.023 |
| Parity | -0.742619 | 0.296543 | 0.476 (0.257-0.827) | 0.012 |
| Gestational age at delivery | 0.002183 | 0.00746 | 1.002 (0.988-1.017) | 0.770 |
| Infant Gender | 0.59188 | 0.109044 | 1.807 (1.462-2.242) | <0.001 |
| TC | -0.418131 | 0.146449 | 0.658 (0.493-0.873) | 0.004 |
| TG | -0.085997 | 0.059986 | 0.918 (0.815-1.029) | 0.152 |
| HDL-C | 0.583509 | 0.13636 | 1.792 (1.371-2.341) | <0.001 |
| LDL-C | 0.391126 | 0.153714 | 1.479 (1.099-2.004) | 0.011 |

Abbreviations: SGA, small for gestational age; TC, total cholesterol; TG, triglycerides; LDL-C/HDL-C, low-density/high density lipoprotein cholesterol-cholesterol.

a: Adjusted for maternal age, race, marital status, gravidity, parity, and infant gender.

| **Table S5** Published studies evaluating the associations between maternal serum lipids and neonatal birth weight (SGA, LGA) | | | | | | | | | | |
| --- | --- | --- | --- | --- | --- | --- | --- | --- | --- | --- |
| Study ID | Study design | Locations | Population(N) | TC | HDL-C | LDL-C | TG | Trimester | Outcomes | Major findings |
| Ye et al.2015 | Prospective observational study | China | non-GDM (n=1243) | √ | √ | √ | √ | 3 | Birthweight, LGA, SGA | HDL-C was the independent protective predictor for LGA; LDL-C, TG were the risk factors of LGA. |
| Vrijkotte et al.2012 | Prospective cohort study | Netherlands | non-GDM (n=4008) | √ |  |  | √ | 1 | LGA, SGA | Elevated TG levels were associated with an increased risk of LGA. |
| Slagjana et al. 2014 | Prospective cohort study | Yugoslavia | GDM (n=200) | √ | √ | √ | √ | 3 | Birthweight, LGA, SGA | TG was higher in the SGA group than in the AGA group; TG was independent predictor for delivering LGA newborns in GDM women. |
| Lei et al.2016 | Prospective cohort study | China | General (n=5535) |  | √ |  | √ | 2 | LGA, SGA | high TG was associated with increased risk of LGA and SGA. |
| Jin et al. 2016 | Prospective cohort study | China | non-GDM (n=934) | √ | √ | √ | √ | 1-3 | LGA, SGA, macrosomia | High HDL-C and relatively low TG were associated with an increased risk of SGA; High TG in late pregnancy was independently associated with increased risks of LGA and macrosomia. |
| Serizawa et al. 2016 | CCS | Japan | non-GDM (n=843) |  | √ | √ | √ | 2 | SGA | LDL-C was a protective factor of SGA. |
| Kramer et al. 2013 | nested CCS | Canada | General (n=994) |  | √ | √ | √ | 2 | SGA | HDL-C was a risk factor of SGA. |
| Ziaei et al. 2012 | CCS | Iran | non-GDM (n=849) | √ | √ | √ | √ | 1 | SGA | Elevated TG were associated with birth weight; No lipids were correlated with SGA. |
| Parlakgumus et al. 2014 | Prospective cohort study | Turkey | non-GDM (n=433) | √ | √ | √ | √ | 1 | LGA, SGA | No lipids were correlated with fetal birth weight, SGA or LGA. |
| Edison et al. 2007 | Prospective cohort study | USA | General (n=1058) | √ |  |  |  | 2 | Birthweight, SGA | Low TC(<3rd percent) was correlated with SGA. |
| Sattar et al. 1999 | cross-sectional study | UK | General (n=16) | √ | √ | √ | √ | ND | IUGR | Women with IUGR pregnancies had lower TC and LDL-C. |
